# Study on the impact of the angular value of scoliosis, the number and lenght of the curves on physical capacity of affected girls

**DOI:** 10.1186/1748-7161-7-S1-O61

**Published:** 2012-01-27

**Authors:** D Czaprowski, T Kotwicki, R Biernat, A Ronikier

**Affiliations:** 1 Faculty of Physiotherapy, Józef Rusiecki University College in Olsztyn, Poland; 2 University of Medical Sciences, Poznań, Poland; 3 Faculty of Rehabilitation, Academy of Physical Education, Warsaw, Poland

## Purpose of the study

To determine the influence of the scoliosis angle, the number and the length of the curves on physical capacity of affected girls.

## Background

Physical capacity determines the organism’s ability to make a physical effort, to tolerate dysfunctions of endogenous homeostasis caused by the physical effort and to quickly regain balance [1][2]. Idiopathic scoliosis (IS) is a systemic disease affecting function of the cardiopulmonary system and impairing patient’s physical capacity [3,5,6].

## Materials and methods

Ninety-seven girls, aged 10 to18, seventy idiopathic scoliosis and 27 controls participated in the study. To determine the physical capacity, the indirect method comprising the PWC170 test was used and maximal oxygen uptake (VO2 max, l/min) was calculated [3,5]. Girls with moderate IS (Cobb 25°-40°) and mild IS (Cobb up to 25°) were analyzed separately.

## Results

The VO_2_ max value (l/min) and the PWC170 index were significantly lower in girls with moderate IS compared to control group. No difference was found between mild IS and controls. No influence of the number of curves and the length of scoliosis on VO_2_ max (l/min; ml/kg/min) and the absolute capacity value (W) was found. A significantly lower value of the PWC 170(W/kg) index was observed in girls with double scoliosis and girls having the curve over 9 vertebrae.

## Conclusions

Girls with moderate IS presented lower VO_2_ max compared to controls. Physical capacity of mild IS was not significantly different from controls. Girls with double scoliosis and girls having the curve over 9 vertebrae had a significantly lower value of the PWC 170 (W/kg) index, moreover no significant effects were found for VO_2_ max and PWC 170 (W).

## References

[B1] Astrand PO Rodahl K Textbook of work physiology 1986 New York: Mc Graw Hill

[B2] Cleland V Wyder T Blizzard L Venn A The provision of compulsory school physical activity: Associations with physical activity, fitness and overweight in childhood and twenty years later Int J Behav Nutr Phys Act 2008 5 14 doi: 10.1186/1479-5868-5-14 10.1186/1479-5868-5-14 18312621 PMC2292742

[B3] Coast JR Cline CC The effect of chest wall restriction on exercise capacity Respirology 2004 9 197 203 10.1111/j.1440-1843.2004.00559.x 15182269

[B4] Durmała J Tomalak W Kotwicki T Function of the respiratory system in patients with idiopathic scoliosis: reasons for impairment and methods of evaluation Stud Health Technol Inform 2008 135 237 245 18401094

[B5] Hawkins MN Raven PB Snell PG Stray-Gundersen J Levine BD Maximal oxygen uptake as a parametric measure of cardiorespiratory capacity Med Sci Sports Exerc 2007 39 103 107 10.1249/01.mss.0000241641.75101.64 17218891

[B6] Koumbourlis AC Scoliosis and the respiratory system Paediatr Respir Rev 2006 7 152 160 10.1016/j.prrv.2006.04.009 16765303

